# A Sequence-Based Damage Identification Method for Composite Rotors by Applying the Kullback–Leibler Divergence, a Two-Sample Kolmogorov–Smirnov Test and a Statistical Hidden Markov Model

**DOI:** 10.3390/e21070690

**Published:** 2019-07-15

**Authors:** Angelos Filippatos, Albert Langkamp, Pawel Kostka, Maik Gude

**Affiliations:** Institute of Lightweight Engineering and Polymer Technology (ILK), Technische Universität Dresden, 01307 Dresden, Germany

**Keywords:** composite rotor, damage identification, structural dynamic behaviour, damage evolution, sequence analysis, hidden Markov model, two-sample Kolmogorov–Smirnov test, Kullback–Leibler divergence

## Abstract

Composite structures undergo a gradual damage evolution from initial inter-fibre cracks to extended damage up to failure. However, most composites could remain in service despite the existence of damage. Prerequisite for a service extension is a reliable and component-specific damage identification. Therefore, a vibration-based damage identification method is presented that takes into consideration the gradual damage behaviour and the resulting changes of the structural dynamic behaviour of composite rotors. These changes are transformed into a sequence of distinct states and used as an input database for three diagnostic models, based on the Kullback–Leibler divergence, the two-sample Kolmogorov–Smirnov test and a statistical hidden Markov model. To identify the present damage state based on the damage-dependent modal properties, a sequence-based diagnostic system has been developed, which estimates the similarity between the present unclassified sequence and obtained sequences of damage-dependent vibration responses. The diagnostic performance evaluation delivers promising results for the further development of the proposed diagnostic method.

## 1. Introduction

Although composite rotors have numerous applications, reliable methods for the on-line damage identification of critical high-performance composite rotors are still at an early stage of development. A number of promising approaches already exist based on vibration-based diagnostics, but their basic disadvantage is a high performance loss due to the existence of ambiguities in the diagnostic data. Investigations demonstrate that such ambiguities in the relationship between the inflicted damage and the resulting change of the modal properties for different damage configurations are most common in composite rotors [1]. As a consequence, a diagnostic feature value corresponds to multiple structural states, and therefore even if the relation between damage state and diagnostic feature can be analytically described, it cannot be inverted. The distinction of two different damage states with similar diagnostic feature values is only possible if past damage events with increasing loads are considered. To achieve this, it is essential to consider past damage events and their effect to the dynamic behaviour as a sequence and not as isolated events.

The development of innovative and efficient damage identification methods is a complex engineering challenge. It requires an interdisciplinary approach that uses simulation and information-based methods, which encompass the material and structural behaviour [2]. In this context, the understanding and analysis of the gradual damage behaviour and of the corresponding structural vibration response of composite rotors plays a central role.

This approach can use diagnostic features acquired from the structural vibration response and aims to identify damage. As damage influences the effective stiffness and damping, it results in a change of the modal properties, i.e., eigenfrequencies, mode shapes and modal damping, which are consequently used for the identification of damage.

### 1.1. Problem and Goal Definition

High-performance composite disc-rotors must withstand complex, inhomogeneous and variable stress fields induced by the centrifugal forces, which are mainly characterised by multi-axial tension. Furthermore, their dynamic response is also affected by the apparent stiffening from the applied centrifugal body forces as well as the complex loading. Consequently, the damage and dynamic behaviour of composite rotors are significantly more complicated than those of stationary composite structures.

In the case of composite materials, failure is typically not considered as a unique event but as a gradual sequence of inter-fibre failure, fibre–matrix de-bonding, delamination and fibre failure. The gradual damage behaviour of composite materials results in changes of stiffness and damping. This is reflected by a measurable gradual change of the dynamic behaviour of the structure. As a result, gradual damage changes different elements in the stiffness and damping matrices, leading to a change in the structural dynamic behaviour.

A common approach in damage identification methods is to conduct isolated structural integrity analyses at different times in the lifetime of the structure [3]. In particular, vibration-based diagnostic methods interpret measurable vibration responses in order to detect, localise and quantify the inflicted damage.

**Figure 1:**
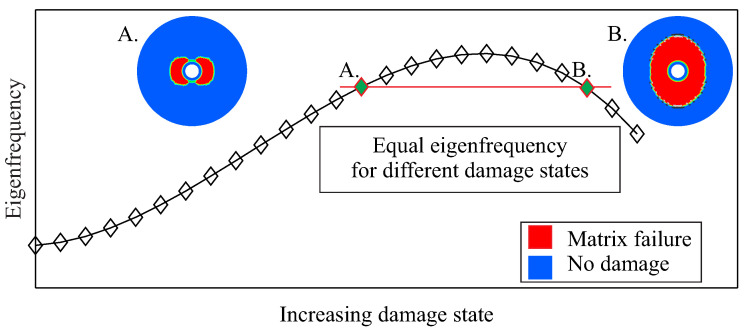
Schematic view of the lack of the bijectivity of the function describing the relation between the state of damage and an eigenfrequency as a diagnostic feature.

A problem encountered is the measurement of equal dynamic properties for different damage configurations, as shown in Figure 1 for a composite rotor. In general, the problem of similar feature values for different damage cases can be described in a mathematical sense as the lack of the bijectivity of the function

(1) Diagnosticfeature=F(Damagestate),

which maps the relation between the state of damage and a damage-dependent feature. Such features normally describe the dynamic behaviour of the structure, e.g.,modal properties. As a consequence, a diagnostic feature value corresponds to multiple discrete structural states, and therefore it cannot always be related to only one damage state.

To overcome this problem, the proposed approach considers previous events in the form of a sequence of features that are sensitive to damage phenomena. Instead of typical analysis of isolated sets of feature values corresponding to different structural states, a sequence is formed from the isolated feature values. This enables the evaluation of the similarity between sequence features collected from the examined rotor and known exemplary sequences.

### 1.2. State-of-the-Art

The development of damage identification methods for composite structures has been a subject of research since the late 1960s according to Doebling [4,5], the period during which Lifshitz and Rotem first proposed the use of vibration measurements for damage detection [6] and were followed by Cawley and Adams [7,8]. Since then, the publications on this topic are notably numerous [3,9,10,11,12,13,14], making a comprehensive overview impracticable in this framework. Instead, the most relevant contributions are merely outlined hereafter.

A common approach for the development of damage identification methods is the application of machine learning algorithms, in which these algorithms are trained to learn datasets containing representative damage configurations [15]. However, in most instances, the generation of such learning sets is not realistic in an experimental way due to prohibitive time and cost factors [16,17]. Therefore, a solution to this problem is the development of reliable simulation models for the generation of learning sets considering only a minimum amount of experimental data for fitting purposes [18].

The implementation of advanced simulation models is a typical approach for damage identification, especially for isolated vibration-based integrity analyses at different time points in the lifetime of the structure according to Farrar, Fassois and Korbicz [3,19,20,21], among others. For example, in previous investigations by Hufenbach and Kostka [14,18,22,23,24], the structural integrity of a carbon fibre-reinforced epoxy plate was investigated where damage was replicated by the attachment of a small mass or a damping layer in order to simulate the alterations of mass and damping characteristics, respectively. In addition, a composite fan blade was investigated and a simple form of a diagnostic approach for damage detection and localisation was implemented in an on-line structural health monitoring system [25]. A problem encountered there was that similar feature values were measured for different positions of the attached mass or for different masses. This problem led to a misclassification of investigated examples. Further investigations performed on a glass fibre-reinforced epoxy disc rotor revealed similar problems [26].

An increase in the diagnostic performance by the solution of the above-mentioned problems could be achieved with the consideration and analysis of isolated damage cases as sequences of damage events, which should be based on an appropriate mathematical theory capable of analysing sequential phenomena [26]. Information theory has been proven to adequately describe and analyse sequences in various research fields by using entropy and the Markov property [27]. Information theory was developed by Shannon to find fundamental limits on signal processing operations such as compressing data and reliably storing and communicating data [28]. Diagnostic methods that consider sequences and implement information-based algorithms for sequence analysis exist in many scientific fields such as medical science, biology and speech recognition [27,29,30]. The concept of entropy as a measure of statistical dependence was introduced by Shannon [28] in a firm mathematical basis, and since then, there has been substantial theoretical development of the concept [31,32,33]. Further substantial investigation based on information theory has been established, including the introduction of some novel algorithms [34,35,36,37]. These algorithms are often based on the sequence similarity estimation for the complete or partial analysis of DNA, protein strings and texts, for their classification to corresponding groups and for the identification of their functionality [38,39]. The development of Markov models and especially hidden Markov models (HMMs) led to their employment in various applications, including speech recognition, target tracking, and protein sequence analysis [27,40].

The application of Markov models in composite materials is mainly focused to the development of damage models under fatigue loading, using acoustic emissions in organic and ceramic matrix composites [41,42,43]. Information-relevant estimators such as entropy have been also applied as a damage detector of isolated damages in composite fibre-reinforced polymer plates [44,45]. However, a sequence analysis of the composite damage and the resulting dynamic behaviour of rotors, compared to the existing classical analysis of isolated events, has not been reported. Furthermore, there is no report of a multi-level diagnostic method considering material and structural behaviour, as well as simulation and information-based methods in order to provide qualitative and quantitative information about the current damage and a prediction of the most probable damage evolution.

### 1.3. Aim and Outline of the Paper

The aim of this work is the development of a multi-level diagnostic system for the identification of the current damage state considering the damage sequence and predict its most probable damage evolution. In Section 2, the most significant aspects are presented and discussed for the consideration of damage evolution as a sequence of events. A novel multi-level diagnostic approach is developed in Section 3 using information theory. Specifically, the multi-level diagnostic approach is developed using the two-sample Kolmogorov–Smirnov test, the Kullback–Leibler divergence and Markov theory as its mathematical basis. Four different diagnostic levels are introduced, each handling a specific problem of damage identification: the detection, the identification of an initial damage from out-of-plane load, the identification of propagating damage from in-plane load and the prediction of the most probable damage evolution.

Considering both experimental and numerical results [1,2], representative damage configurations are considered for the generation of input data to be utilised for the multi-level diagnostic model. The corresponding damage and vibration response sequences are formed to a diagnostic database in Section 4 and used as diagnostic input data. Then, having as input the diagnostic database, the similarity between the available sequences collected on an examined composite rotor and on known exemplary sequences are evaluated in Section 5.1. Subsequently, a performance evaluation of the approach is conducted, results are presented in Section 5.2 for every identification level, the classification accuracy is calculated and conclusions are discussed.

## 2. Aspects for the Consideration of Damage Evolution as A Sequence of Events

In the case of composite rotors, damage does not necessary cause immediate total failure, which would be typical of metallic rotors, but rather it manifests itself as an increase of micro-crack density and delamination-growth resulting in local changes of stiffness and damping. This leads to noticeable alterations of the structural dynamic behaviour in a sequential way.

As the damage evolution of composite materials can be modelled as a continuous process, it can be discretised to *m* states and considered as a sequence of these distinct states [26]. For each state, simulation models combined with damage mechanics models can also provide the damage-dependent structural dynamic behaviour [2]. In this way, two sequences with inter-dependent elements can be formed, a damage evolution sequence and a corresponding dynamic response sequence. The damage evolution sequence includes distinct damage states and the dynamic response sequence includes the corresponding dynamic responses.

### 2.1. Damage Evolution Sequence of Composites

Different damage states of composite rotors can cause non-monotonic changes in the dynamic behaviour, which result in similar diagnostic feature values when only isolated damage events are considered [1]. Therefore, an isolated vibration-based diagnostic approach is not sufficient and an extended approach is necessary. To achieve a reliable and unique diagnostic statement, a sequence-based diagnostic model is essential, which considers past damage states and the corresponding dynamic behaviour as a damage sequence, and not as isolated states [26].

Based on sequence analysis, a multi-level diagnostic system is developed here in order to first detect damage and then identify damage from out-of-plane and in-plane loading. The goal of this multi-level diagnostic system is to identify the most probable damage evolution of composite rotors based on the determined vibration responses under unknown load conditions.

**Figure 2:**
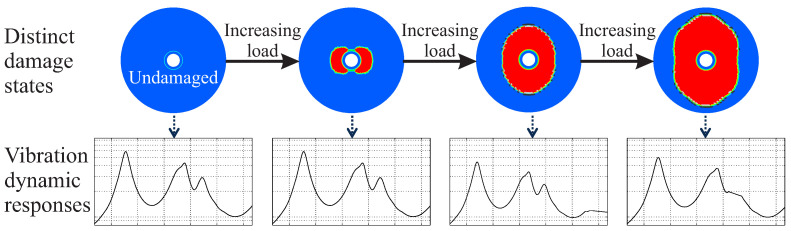
Illustration of the damage evolution sequence and the corresponding dynamic response sequence of a composite disc-rotor.

A composite rotor of state $h(S_{0})$ undergoes an unknown loading that causes a damage initiation and propagation. Due to this event, the rotor has a new structural state $h(S_{1})$. Consequently, under increasing loading resulting in damage propagation, the rotor undergoes multiple states from $h(S_{0})$ to $h(S_{m})$, as shown in Figure 2.

A **damage evolution sequence** (DES) of a composite rotor is then defined as an arbitrary sequence DES of *m* discrete damage states, with

(2) $$DES=\langle S_{1},S_{2},\ldots ,S_{i},\ldots ,S_{m}\rangle .$$

Each term $S_{i}$ of the damage evolution sequence DES from Equation (2) is a matrix, which corresponds to a damage state of the rotor [26]. Every state term $S_{i}$, describes the existence of a failure mode at *K* separated representative elements of the rotor. The number of elements *K*, in the case of a finite element analysis, is the number of the meshed elements. Based on the previously described damage mechanics model, five failure modes are considered, three different matrix failure types, IFF1, IFF2 and IFF3, and two fibre failure types FF1 and FF2 [46], thus each $S_{i}$ has a size of K×5,

(3) $$S_{i}=\left\{\begin{array}{ccccc} FF1_{1} & FF2_{1} & IFF1_{1} & IFF2_{1} & IFF3_{1}\\ FF1_{2} & FF2_{2} & IFF1_{2} & IFF2_{2} & IFF3_{2}\\ \vdots & \vdots & \vdots & \vdots & \vdots \\ FF1_{K} & FF2_{K} & IFF1_{K} & IFF2_{K} & IFF3_{K} \end{array}\right\}.$$

These parameters could describe, e.g., the position and size of an impact-caused initial damage. Such parameters are extremely important and can be treated as labels, for example at the performance estimation of damage identification algorithms [15].

### 2.2. Dynamic Response Sequence of Composites

The structural dynamic behaviour is then considered in a similar way. In particular, the terms of the eigenfrequency and the modal damping ratio include damage-relevant information. The damage-dependent dynamic behaviour of a rotor during its lifetime forms a **dynamic response sequence** DRS with

(4) $$DRS=\langle E_{1},E_{2},\ldots ,E_{i},\ldots ,E_{m}\rangle ,$$

where each state $E_{i}$ of the dynamic response sequence is a vector including *p*-length information of the structural vibration response,

(5) $$E_{i}=\left\{P_{yy,i}^{1},P_{yy,i}^{2},\ldots ,P_{yy,i}^{p}\right\}.$$

In this way, each dynamic state $E_{i}$ of the dynamic response sequence DRS corresponds to a damage state $S_{i}$ of the damage evolution sequence DES [26].

#### 2.2.1. Structural Vibration Response in Time Domain

**Figure 3:**
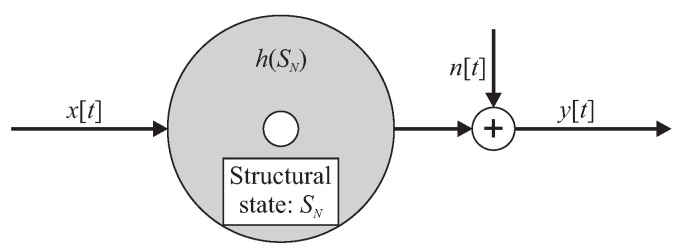
Representation of a time-invariant rotor with excitation signal x[t], response signal y[t], and additive noise n[t].

A rotor *h* with a structural state $S_{N}$, as shown in Figure 3, is subjected to an excitation signal x[t] and generates a vibration response signal y[t] corrupted with additive noise n[t] for t=1,2,...,K discrete time samples with a sampling frequency of $f_{s}$.

Then, the vibration system of the rotor can be described as [47]:

(6) $$y\left[t\right]=\sum_{\tau =0}^{\infty }h\left(S_{0}\right)\left[\tau \right]\cdot x\left[t-\tau \right]+n\left[t\right].$$

If the magnitude of the excitation force acting on the rotor is known, the excitation is viewed as deterministic. Then, the magnitude and frequency of the excitation at any future time can be completely predicted from the signal history, and the resulting vibration is viewed as a deterministic vibration. However, if the magnitude of the excitation is unknown for all time, and only averages and deviations are known, the excitation is said to be random. For the case of random excitation, which often holds for composite rotors, the response is also random and cannot be determined exactly in any instant of time. The rotor vibration response can be determined only in terms of statistical quantities such as the mean and the mean square values of the excitation and only the probability of the occurrence of designated magnitudes and frequencies can be predicted [48]. Randomness, therefore, is a characteristic of the excitation and the vibration, and not of the mode shapes or the eigenfrequencies of the rotor.

In vibration theory, random vibration can be assumed as an ergodic and stationary random process. A deterministic vibration is a special case of a dynamic process, and can be viewed as a random vibration [49].

A random process *Y*, generating a random vibration *y*, is wide-sense stationary if the mean function $\mu_{Y}$ and autocorrelation function $R_{YY}$ are invariant to a time shift.

(7) $$\begin{array}{c} \mu_{Y}\left(t\right)=\mu_{Y}=constant,\\ R_{YY}\left(t,t+\tau \right)=R_{YY}\left(\tau \right). \end{array}$$

The autocorrelation function $R_{YY}\left(\tau \right)$ can be determined as

(8) $$R_{YY}\left(\tau \right)=\sum_{t=-\infty }^{\infty }E\left[y(t)y(t+\tau )\right].$$

A wide-sense stationary random vibration response is ergodic, if the time average of one sequence of events is the same as the ensemble average E,

(9) $$\begin{array}{cc} \langle y(t)\rangle & =E[Y(t)],\\ \langle y(t+\tau )\rangle & =E[Y(t)Y(t+\tau )]. \end{array}$$

A wide-sense stationary and ergodic random vibration signal y(t) does not have finite energy and therefore a discrete-time Fourier transform cannot be applied. However, it possesses a finite average power $P_{avg}$ of overall time, which is calculated by the following time average divided by its period *T* as the period approaches infinity:

(10) $$P_{avg}=\lim_{T\to \infty }\frac{1}{2T}\int_{-T}^{T}y{\left(t\right)}^{2}\;dt.$$

#### 2.2.2. Power Spectral Density Estimation

Time domain analysis processes the data over a time period and provides the behaviour of the signal over time. This type of analysis allows predictions and regression models for the vibration in the time domain. However, as in most cases, a random signal appears as a complex waveform in the time domain, it is not an easy task to characterise and analyse the time signal [50].

On the other hand, frequency domain analysis is applied when processes such as signal filtering, amplifying and mixing are required. The description of a signal by its frequency contents is appealing both intuitively and from an engineering point of view according to Ljung [51]. By using frequency domain analysis, it is possible to identify the dynamic characteristics of the structure, which in turn can be used for the structural assessment and for damage identification purposes.

Random vibration can be represented in the frequency domain by a power spectral density (PSD) function. It estimates the root-mean-square (RMS) value of some magnitude response, divided by the vibration frequency. The RMS value of a signal is equal to the standard deviation σ, assuming a zero mean, which is the case for a wide-sense stationary ergodic vibration response [52]. Thus, PSD defines the distribution of power over the frequency range of excitation, and provides information about the statistical properties of the signal in the frequency domain [53]. A power spectral density can be calculated for any type of vibration signal (deterministic or random), but it is particularly appropriate for random vibration [52].

The Fourier transform Y(f) of a continuous random response y(t) is:

(11) $$Y\left(f\right)=\int_{-\infty }^{\infty }y\left(t\right)e^{-i2\pi ft}dt\;,\;\mathrm{where}\;-\infty <f<\infty .$$

A typical non-parametric approach to calculate the PSD $S_{YY}$ is taking the limit of the Fourier transform Y(f) to its complex conjugate $Y^{*}\left(f\right)$ divided by its period T as the period approaches infinity [52],

(12) $$S_{YY}\left(f\right)=\lim_{T\to \infty }\frac{1}{T}Y\left(f\right)Y^{*}\left(f\right).$$

The power spectral density function can be estimated using different methods, both non-parametric and parametric [54]. A typical approach is to take the discrete-time Fourier transform of the autocorrelation function, which is the Wiener–Khintchine approach. The above approach to estimate a PSD is also applied to discrete time vibration responses $y_{n}$. However, only an estimation of the true PSD can be calculated, because the true PSD can be determined only for an infinite amount of time samples.

A finite window is considered with 1≤n≤N and a sampled signal at discrete times $y_{n}$. Then, for a discrete-time process $Y_{n}$, an estimate of the PSD can be obtained by the discrete-time Fourier transform of its autocorrelation sequence,

(13) $$P_{yy}\left(f\right)=\sum_{n=1}^{N}R_{YY}\left(n\right)e^{-i2\pi fn},$$

whereas the true PSD is achieved when *N* approaches infinity.

#### 2.2.3. Applications of Spectral Methods for Probability Distribution Estimation

In the estimation of the PSD of a wide-sense stationary and ergodic random vibration response, the spectral features can be estimated only approximately with a finite amount, *T*, of data. The true PSD can only be calculated in the abstract limit T→∞. Therefore, the exact description of the PSD of a random vibration requires an abstract mathematical model for the data, the limit spectrum shown in Equation (12) [55]. As already mentioned, the limit spectrum can only be approximately inferred with the use of a finite amount of a data from an existing dataset, which leads to the concept of statistical inference and the application of a probabilistic approach. Therefore, an estimated PSD is a power distribution over a frequency range for a given data sample of a statistical population. Subsequently, each frequency range Δf has a probable power percentage over the whole finite average power $P_{avg}$ from Equation (10), given a finite amount, *T*, of data sample.

Previous investigations have already shown that the power spectral density of a discrete-time stationary random process has similar properties to a probability density function (PDF) of a continuous random variable: both are non-negative and have a finite area [56]. This enables the application of the well-known spectral estimation methods to problems that involve and require probability distributions. For example, the aforementioned approach is applied by Zamora and Kay [57,58,59] for a PDF estimation from random variables. Similarly, different parametric and non-parametric PSD estimators are applied for an entropy estimation of a continuous random variable [56,60]. Applications of spectral estimation methods are also reported for the detection and classification of surface chemicals using parametric autoregressive models from Ding [61].

Furthermore, a PSD comparison based on statistical hypothesis using different statistics has been applied, namely, the Kolmogorov–Smirnov type statistic, the $\chi^{2}$ statistic, the Kullback–Leibler type statistic and the $L_{2}$ distance [62,63]. In addition, Georgiou [64] applied a pseudo Riemannian metric to calculate distances of PSDs from different random processes for a prediction problem.

To use spectral estimation methods for probability distribution estimation, the PSD is normalised by the total integrated power with a sum equal to unity. A normalised estimated PSD, denoted as $P_{yy}^{{}^{\prime }}$, is then

(14) $$P_{yy}^{{}^{\prime }}\left(f\right)=\frac{P_{yy}\left(f\right)}{\sum_{f=f_{1}}^{f_{2}}P_{yy}\left(f\right)},$$

for $f=[f_{1},f_{2}]$ selected frequencies of interest.

## 3. Sequence-Based Diagnostic System for Damage Identification

Based on sequence analysis, a multi-level diagnostic system is developed here in order to first detect damage and then identify damage from multi-axial loading. The goal of this multi-level diagnostic system is to identify the most probable damage evolution of composite rotors based on the determined vibration responses under unknown load conditions.

### 3.1. Synthesis of Sequence-Based Diagnostic System

**Figure 4:**
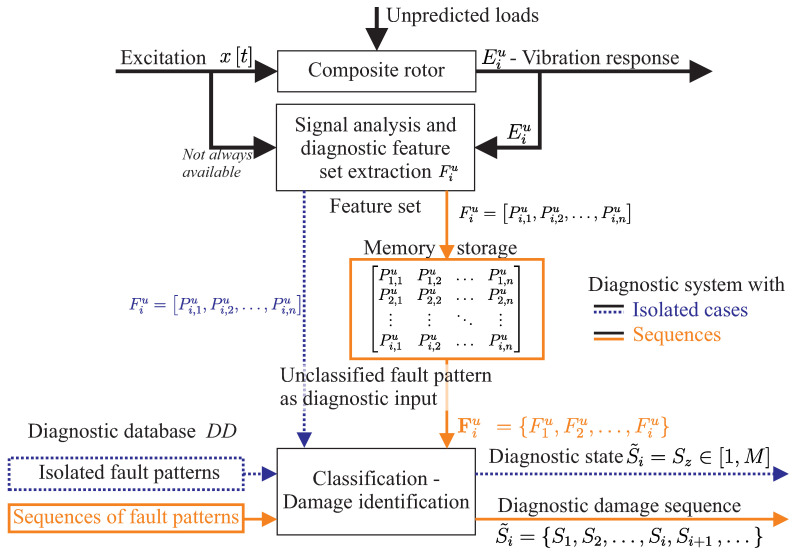
Sequence-based diagnostic system as a classification of fault patterns based on the idea from Korbicz [19], as an extension for the consideration of the gradual damage behaviour of composite rotors.

The sequence-based system developed here extends an information-based diagnostic system from Korbicz [19], which identifies damage only from isolated fault patterns, as shown in Figure 4. However, the diagnostic system developed here predicts damage by matching the currently determined vibration response sequence to previously measured and calculated vibration response sequences.

For the prediction of the damage state of the rotor, the damage from both the out-of-plane and the in-plane loading has to be identified. This is because the introduced loading diversely affects the gradual damage behaviour of composite rotors up to final failure. Consequently, through the acquired information about the damage state of the rotor, a prediction of the most probable damage evolution is conducted.

To identify the damage occurring from out-of-plane compression loads as well as from in-plane loads due to rotor run-up, a multi-level diagnostic system is developed with four different diagnostic levels, similar to the approach of Rytter [65]:

- Diagnostic Level 1: Damage detection
- Diagnostic Level 2: Identification of initial damage from out-of-plane load
- Diagnostic Level 3: Identification of propagating damage from in-plane load
- Diagnostic Level 4: Prediction of most probable damage evolution

**Figure 5:**
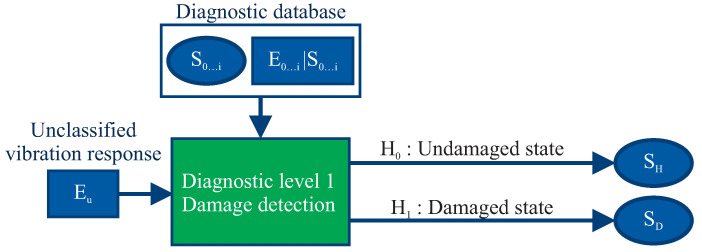
A diagnostic model using binary statistical hypothesis testing for damage detection, having as input a diagnostic database $\left\{S_{i},E_{i}|S_{i}\right\}$ and an unclassified vibration response $E_{u}$.

For Diagnostic Level 1, a diagnostic model is formed in Section 3.2 based on a binary statistical hypothesis testing using experimental data from the diagnostic database, as shown in Figure 5.

Once a more specific analysis of the detected damage is required, the diagnostic model for Level 1 ceases to work. For the damage identification and prediction, prior knowledge of the dynamic behaviour for each of the expected damage states is required as well as a detailed description. As this is usually not possible in an experimental way, simulation data from the diagnostic database are used for the remaining diagnostic levels.

For Diagnostic Levels 2–4, a diagnostic model is developed based on the Markov process, as shown in Section 3.3. Specifically, a hidden Markov model is formed from the simulation data of the diagnostic database.

**Figure 6:**
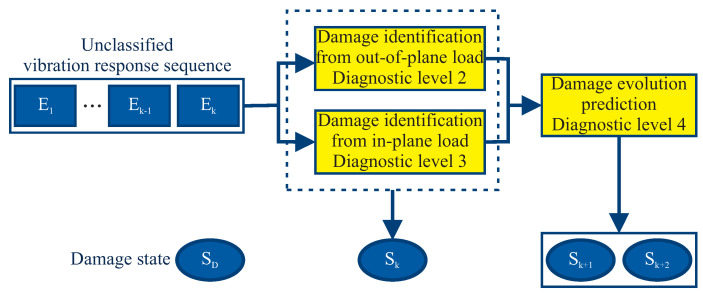
A multi-level diagnostic system, having as input an unclassified vibration response sequence $E_{1},\ldots ,E_{k-1},E_{k}$.

At Levels 2–4, the input of the diagnostic model is a sequence of unclassified vibration responses $E_{1},\ldots ,E_{k-1},E_{k}$, as shown in Figure 6. The response sequence is compared with each vibration response sequence $E_{1},\ldots ,E_{N}$ in order to find the most similar one. To achieve this, the Viterbi algorithm is applied, which is a dynamic programming algorithm for finding the most likely sequence of hidden states ${\tilde{S}}_{i}$ given a sequence of observed responses $E_{0\ldots N}$. Finally, for Diagnostic Level 4, a prediction for every output ${\tilde{S}}_{i}$ is provided from the diagnostic model in the form $\left\{S_{k+1},S_{k+2},S_{\ldots }\right\}$.

### 3.2. Selection of Diagnostic Model for Damage Detection

Damage detection does not require prior knowledge of the dynamic behaviour for all possible expected damage states, but rather a well-defined feature space of the undamaged state, under the investigated operational conditions. Therefore, damage can be detected from experimental data, and each unclassified vibration response is detected as an outlier of the defined space and is considered as a response from a damaged state.

Assuming that after a time period t+τ the rotor $h[S_{0}]$ with a state $S_{0}$ has been transformed to $h[S_{u}]$ with a state $S_{u}$ due to a load event, the statistically same excitation signal x[t] generates a vibration response signal $E^{{}^{\prime }}\left[t\right]$ where

(15) $$E\left[t\right]\neq E^{{}^{\prime }}\left[t+\tau \right],$$

which denotes two statistically different dynamic responses due to the introduced change in the rotor. The statistical comparison of this difference is used as a damage indicator, and a binary statistical hypothesis test is formulated with two different statistical measures in order to detect damage. Specifically, two statistical measures are investigated, the two-sample Kolmogorov–Smirnov test and the Kullback–Leibler divergence, and subsequently their diagnostic accuracy is studied in Section 5.1.

#### 3.2.1. Formulation of Binary Statistical Hypothesis Test

**Figure 7:**
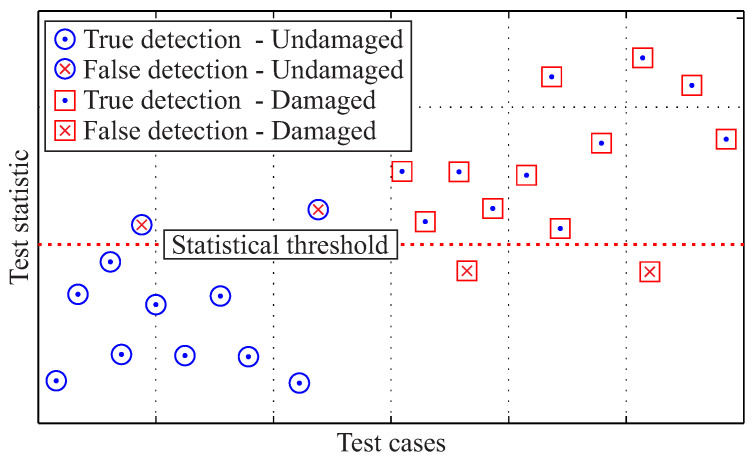
Illustration example of classified intact and damage test cases under the null hypothesis using a statistical threshold.

Damage detection is based on a proper comparison of the unclassified vibration response $E_{u}$ to the undamaged response $E_{0}$ via a binary statistical hypothesis test. The proposed statistical test uses the dynamic responses $\left\{E_{0}|S_{0}\right\}$ and $\left\{E_{u}|S_{u}\right\}$ and provides a proper relationship, such as equality or inequality. The hypothesis testing problem is then formulated as

(16) $$H_{0}:\left\{E_{u}|S_{u}\right\}=\left\{E_{0}|S_{0}\right\},$$

which is the null hypothesis, that $S_{u}=S_{0}$, equal to the undamaged state of the rotor and

(17) $$H_{1}:\left\{E_{u}|S_{u}\right\}\neq \left\{E_{0}|S_{0}\right\},$$

which is the alternative hypothesis, i.e., $S_{u}\neq S_{0}$, in this case that the rotor is damaged. Then, for the decision under the null hypothesis $H_{0}$, a threshold *l* is computed based on the statistical measure and evaluated for the given experimental data and for each rotor separately. In order for the null hypothesis $H_{0}$ to be accepted, the value of the test statistic $F_{Test}\left(E_{0},E_{u}\right)$ for the investigated vibration responses should be smaller or equal to the computed threshold *l*,

(18) $$\begin{array}{c} F_{Test}\left(E_{0},E_{u}\right)\leq l⟹H_{0}\;\mathrm{is}\;\mathrm{accepted}\;(\mathrm{undamaged}\;\mathrm{rotor})\;\\ ⟹H_{1}\;\mathrm{is}\;\mathrm{accepted}\;(\mathrm{damaged}\;\mathrm{rotor}),\; \end{array}$$

as illustrated in Figure 7, and with a confidence bound, which contains at least 95% of the undamaged cases.

#### 3.2.2. Kolmogorov–Smirnov Test

The two-sample Kolmogorov–Smirnov test (KS${}^{2}$-Test) is selected as it is a non-parametric hypothesis test that evaluates the difference between the empirical distribution functions of two cumulative distribution functions (CDFs) from different probability distributions [66,67,68]. It uses as input two sample data vectors generated from probability distributions, and the test statistic is formulated as

(19) $$F_{Test}\left(E_{0},E_{u}\right)=\underset{x}{\sup}\left|CDF_{0,n}\left(x\right)-CDF_{u,n^{\prime }}\left(x\right)\right|,$$

where $CDF_{u,n}$ and $CDF_{0,n^{\prime }}$ are the empirical distribution functions of the undamaged and the unclassified vibration responses, respectively, and sup is the supremum function. The null hypothesis is rejected at the significance level α of the hypothesis test if

(20) $$F_{Test}\left(E_{0},E_{u}\right)>c\left(\alpha \right)\sqrt{\frac{n+n^{\prime }}{nn^{\prime }}},$$

where *n* and $n^{\prime }$ are the sizes of first and second sample, respectively, and the value of c(α) for each significance level of α can be retrieved from Miller [69].

#### 3.2.3. Kullback–Leibler Divergence

The Kullback–Leibler divergence $D_{KL}$ is selected as it is a statistical measure of the difference between two probability distributions $P_{yy}$ and $Q_{yy}$, and is denoted as

(21) $$D_{KL}(P_{yy}\left|\right|Q_{yy}).$$

It can be used for damage detection in composite rotors as a measure of the disorder between two vibration responses in the frequency domain. For discrete probability distributions, $D_{KL}$ is defined as

(22) $$D_{KL}(P_{yy}\left|\right|Q_{yy})=\sum_{i}P_{yy}\left(i\right)log_{b}\frac{P_{yy}\left(i\right)}{Q_{yy}\left(i\right)},$$

where *b* is the base of the logarithm used and the unit of $D_{KL}$ is bit for b=2. The value of the test statistic $F(E_{0},E_{u})$ for the investigated vibration responses is then calculated and the null hypothesis is accepted if

(23) $$F\left(E_{0},E_{u}\right)=D_{KL}(E_{0}\left|\right|E_{u})\leq l$$

for a given confidence bound *l*, which contains at least 95% of the undamaged cases.

### 3.3. Development of Diagnostic Model for Damage Identification and Prediction

The proposed diagnostic model for damage identification and prediction is based on a phenomenological approach, which models the gradual damage behaviour in a form of discrete Markov chains. For a valid implementation of this approach within the framework of Markov theory, the following four criteria should be fulfilled. The most fundamental one requires that the gradual damage behaviour follows a Markovian type of evolution, i.e., it exhibits the Markov property. This means that the future damage state depends only on the present damage state and on the applied loads. This is intuitive and has also been verified and applied in a number of investigations [42,70,71,72]. Furthermore, the rotor is subjected to loads which are discrete and describe different loading conditions. In addition, the damage increases monotonically under increasing loading and has distinct damage states similar to the work done by Pappas [43].

**Figure 8:**
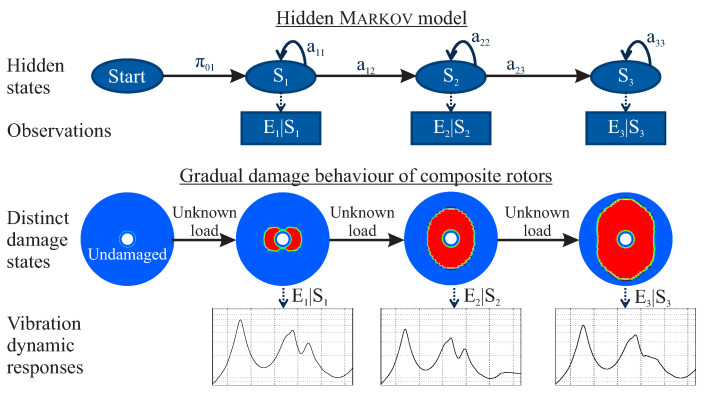
Illustration of a statistical hidden Markov model and of the gradual damage behaviour of composite rotors.

Based on the Markov property, it is possible to create a Markov model where two different and inter-dependent sequences exist. The first of these sequences cannot be observed but can be identified based on the observation of the second sequence, as shown in Figure 8. Such model is called a Hidden Markov Model (HMM). In a HMM, the state is not directly visible, but the output called emission, dependent on the state, is visible. Each unobserved state has a probability distribution over the possible output tokens. The tokens are possible values that can be emitted from the HMM.

As composite damage is not directly visible and cannot be observed in most cases during operation, a damage state of a composite rotor can be considered as an unobserved hidden state of a Markov process. Furthermore, the dynamic behaviour of each damage state can be observed, and this behaviour is directly dependent upon the damage state itself. Therefore, the dynamic behaviour of a composite rotor at different damage states can be considered as an observation of a Markov process.

A hidden Markov model is formally described as a 5-tuple [27],

(24) ϑ=(n,m,A,B,π),

where *n* is the number of distinct states, *m* is the number of distinct observations at each state, *A* is the transition matrix, *B* is the observation emission matrix and π is the initial probability vector. A HMM has two main parts. The first part includes a Markov chain $S_{i}$, represented by a statistical matrix $A_{n\times n}$, which describes the transition probabilities between the *n* states of the model, together with an initial probability distribution π, where $\pi_{i}=p\left(S_{1}^{u}=S_{i}\right),1\leq i\leq n$.

**Figure 9:**
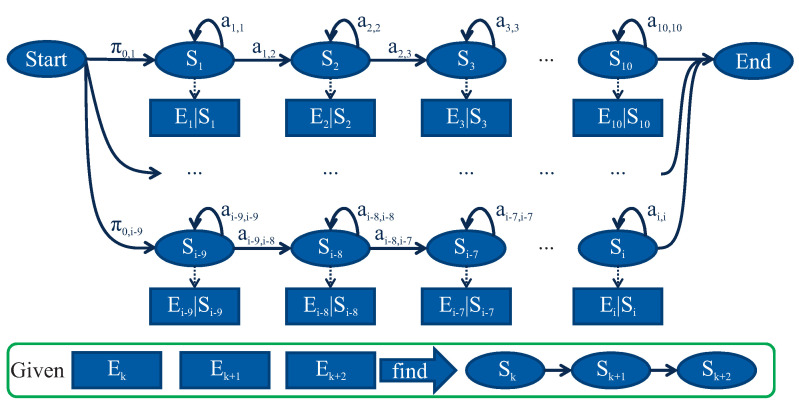
Illustration of the developed statistical HMM.

The second part consists of a set of probability distributions, one for each hidden state, which model the emission of the observations. If there are *m* possible distinct observations, then the probability distributions $b_{i}$ form a matrix $B_{n\times m}$. An example of the developed statistical HMM is shown in Figure 9.

#### 3.3.1. Distinct States

The diagnostic database comprises *n* damage states, which are denoted as

(25) $$S=\left\{S_{1},S_{2},...,S_{n}\right\}.$$

Each HMM state corresponds to a damage state of the rotor and contains information regarding the failure modes, the damage extent and the classifying labels.

#### 3.3.2. Distinct Observations

For each damage state $S_{i}$, there are *m* distinct observations. In the case of a vibration response, the normalised PSD is partitioned into *m* subsections, where *m* is the length of the frequency response, i.e., the number of FFT lines, for $f=[f_{1},f_{2}]$ selected frequencies. Thus, each frequency value is considered as a distinct observation *i* and its magnitude is considered to be its probability $p_{i}$.

#### 3.3.3. Observation Matrix

Based on the number of distinct observations and the number of damage states, the observation emission matrix $B_{n\times m}$ is formed. Each row $B_{i,j}$ contains the vibration response $E_{i}$ in the form of a probability distribution for each damage state $S_{i}$. B(k,l) is the probability that the symbol *l* is emitted from a state *k*,

(26) $$B=\begin{array}{cc} & \begin{array}{ccccc} f_{1} & f_{2} & f_{3} & \;\cdots & f_{m} \end{array}\\ \begin{array}{c} S_{1}\\ S_{2}\\ S_{3}\\ \vdots \\ S_{n} \end{array} & \left(\begin{array}{ccccc} b_{1,1} & b_{1,2} & b_{1,3} & \cdots & b_{1,m}\\ b_{2,2} & b_{2,2} & b_{2,3} & \cdots & b_{2,m}\\ b_{3,1} & b_{3,2} & b_{3,3} & \cdots & b_{3,m}\\ \vdots & \vdots & \vdots & \ddots & \vdots \\ b_{n,1} & b_{n,2} & b_{n,3} & \cdots & b_{n,m} \end{array}\right) \end{array}.$$

As every row of the observation matrix *B* includes a probability distribution, it holds true that

(27) $$\sum_{j=1}^{m}b_{i,j}=1\;for\;i\in \left[1,n\right].$$

As the developed HMM requires a discrete symbol set for the elements of the observation vector, a vector quantisation is applied based on Lloyd’s algorithm, which maps the continuous observation vector into a discrete codebook index [27,73]. As each distinct state $S_{i}$ has a specific probability distribution $P_{yy,i}$, a data sample ds is generated with a code length cl. This data sample is then emitted from the respective distinct state, and it holds true that

(28) $$B\left(S_{i}\right)=p\left(ds_{i}\right)\;for\;i\in \left[1,n\right].$$

#### 3.3.4. Transition Matrix

The transition probabilities for each damage state form a transition matrix $A_{n\times n}$. The transition probabilities control the way the hidden state at a specific step *k* is chosen, given the hidden state at step k−1. The state’s transition probability distribution is denoted as $A=a_{i,j}$, where $a_{i,j}$ is the probability of transition from state *i* to state *j*. In the case where any state could reach any other state in a single step, the matrix *A* would be a full matrix with $a_{i,j}>0$. In the current work, the transition matrix is formulated based on the investigated damage scenarios and has a similar form to a one-state left–right hidden Markov model [27]. The state transition matrix is in the following form:

(29) $$A=\begin{array}{cc} & \begin{array}{ccccc} S_{1} & S_{2} & S_{3} & \;\cdots & S_{n} \end{array}\\ \begin{array}{c} S_{1}\\ S_{2}\\ S_{3}\\ \vdots \\ S_{n} \end{array} & \left(\begin{array}{ccccc} a_{1,1} & a_{1,2} & a_{1,3} & \cdots & a_{1,n}\\ a_{2,2} & a_{2,2} & a_{2,3} & \cdots & a_{2,n}\\ a_{3,1} & a_{3,2} & a_{3,3} & \cdots & a_{3,n}\\ \vdots & \vdots & \vdots & \ddots & \vdots \\ a_{n,1} & a_{n,2} & a_{n,3} & \cdots & a_{n,n} \end{array}\right) \end{array}.$$

The transition probabilities on the diagonal $a_{i,j},i=j$ give the probability that the model remains in the same state. Under the assumption that the model receives a constant length of data sample values for each unclassified vibration response, the probability is set to

(30) $$a_{i,j}=\frac{1}{\left|ds\right|}\;\mathrm{for}\;i=j.$$

for the remaining transition probabilities where $a_{i,j},i\neq j$, the following approach is adopted. For the transitions that are physically impossible, a zero probability is given,

(31) $$a_{i,j}=0\;\mathrm{for}\;i\neq j\cap Dam_{j}<Dam_{i},$$

meaning that there can be no transition from a state to another, through which damage would be reduced. The remaining transition probabilities from one to another state with increased damage are then calculated as

(32) $$a_{i,j}=1-\frac{1}{\left|ds\right|\cdot \left(m-1\right)}\;\mathrm{for}\;i\neq j.$$

for every row of the transition matrix *A*, it holds true that

(33) $$\sum_{i=1}^{n}a_{i,j}=1\;\mathrm{for}\;j\in \left[1,n\right].$$

#### 3.3.5. Initial Probability Vector

The probability that the initial state can be selected among many possible initial damage states is denoted as π,

(34) $$\pi =\begin{array}{cc} & \begin{array}{ccccc} S_{1}\; & S_{2}\; & S_{3}\; & \;\cdots & S_{n} \end{array}\\ S_{0} & \left(\begin{array}{ccccc} \pi_{0,1} & \pi_{0,2} & \pi_{0,3} & \cdots & \pi_{0,n} \end{array}\right) \end{array},$$

where

(35) $$\pi_{0,i}=\frac{1}{n},$$

for every $\pi_{0,i}$ and an initial probability vector of length *n*. In the current investigations, an equal probability $\pi_{0,i}$ exists for each damage state of the investigated damage sequences.

#### 3.3.6. Similarity Analysis of an Unclassified Vibration Response Sequence

After having formulated the HMM as a 5-tuple, the diagnostic goal is to compare an unclassified vibration response sequence $E^{u}$ with classified vibration response sequences, in order to find the most similar sequence of damage states,

(36) $$\left[E_{1}^{u},E_{2}^{u},\ldots ,E_{k}^{u}\right]|HMM\overset{find}{\to }\left[S_{1},S_{2},\ldots ,S_{k}\right].$$

To achieve this, the Viterbi algorithm is applied, which is a dynamic programming algorithm for finding the most likely sequence of hidden states from a sequence of observed events [27]. Given an unknown sequence of emissions, it calculates the most likely state path through the hidden Markov model with the goal of finding a state sequence with the highest calculated posterior state probability. The diagnostic results from such a similarity analysis are presented in Chapter Section 5 for multiple cases of the investigated rotor.

## 4. Case Study of A Composite Disc Rotor

The selected fibre architecture is composed of a glass-fibre, non-crimp, multi-ply and multi-axial fabric (NCF) [74,75]. The composite lay-up consists of four such fabrics $[0^{\circ }/-45^{\circ }/90^{\circ }/+45^{\circ }/-45^{\circ }/90^{\circ }$$/45^{\circ }/0^{\circ }/0^{\circ }/-45^{\circ }/90^{\circ }/45^{\circ }/-45^{\circ }/90^{\circ }/45^{\circ }/0^{\circ }]$, resulting in a total laminate thickness of 4 mm. The inner and outer diameter of the rotor are 60 mm and 500 mm, respectively [1]. The lay-up results in an in-plane orthotropic behaviour, and it is selected in order to achieve a polar non-symmetrical damage evolution caused by applied rotational loads. This material was extensively investigated in previous research projects, where the results indicated a gradual damage behaviour [76].

**Figure 10:**
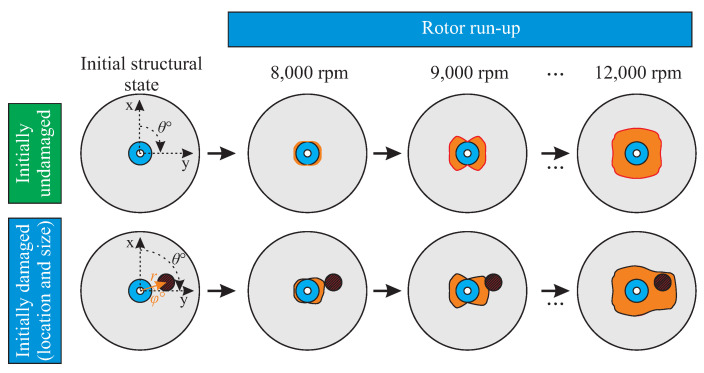
Illustration of two different damage sequences resulting from damage propagation during multiple rotor run-ups: one sequence without any initial damage (**top**), and one having an initial damage due to an out-of-plane loading (**bottom**).

The current approach utilises and further develops previous investigations [24,77] by specifying different load events, as it is shown principally in Figure 10:

- An out-of-plane load is introduced, resembling an impact event, which results to an initial damage at different locations and with different load levels.
- The composite rotor is run up to different rotational velocities, in order to induce further damage from increasing in-plane centrifugal loading.

Using such a combination of out-of-plane and in-plane loads, representative damage sequences were generated with multiple structural states, for which their damage state and their dynamic behaviour was investigated [1,78]. There, a sequence without initial damage was investigated at three nominally equal rotors, in order to investigate possible scatter of the damage sequence. Furthermore, two different sequences were tested with the same initial damage at two different positions of $0^{\circ }$ and $90^{\circ }$ by a radius of 100 mm, in order to investigate the effect of the position of the initial damage on the damage sequence. The gained data are used here as test data in Section 5.1.

### 4.1. Generation of Input Data for The Diagnostic Database

To systematically generate different damage configurations, the full factorial design of numerical experiments was implemented for the simulation study, presented in previous investigations [2]. The loading parameters were set as *n* factors, factor, and are assigned a discrete set of levels. A generated sample, using the full factorial design, was a *n*-dimensional space of damage configurations and contained all possible combinations of the set of factors. The sample size was calculated as the product of the numbers of levels of the factors,

(37) $$\left|Sample\right|=\prod_{i=1}^{n}factor_{i}.$$

**Figure 11:**
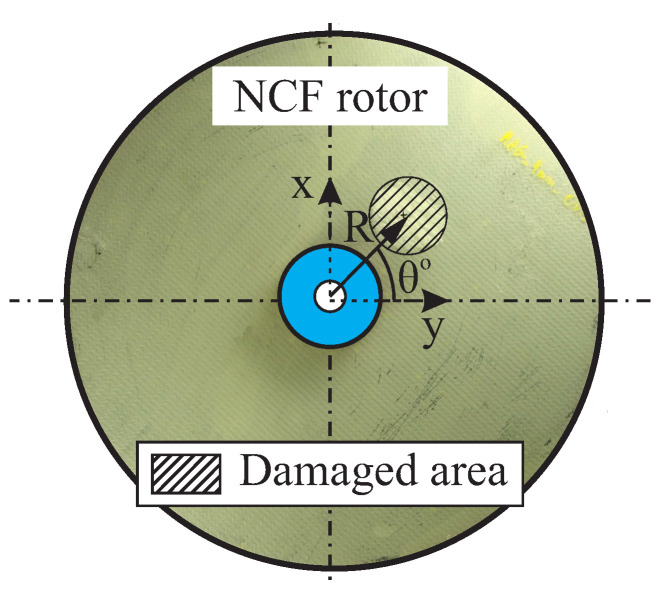
Position parameters (R,θ) for an out-of-plane load for an Cartesian-orthotropic rotor.

The magnitude and position of the simulated out-of-plane loads were selected as factors, as shown in Figure 11, in order to create different test cases. The position of the out-of-plane load was defined by the radius *R* and the angle θ.

**Figure 12:**
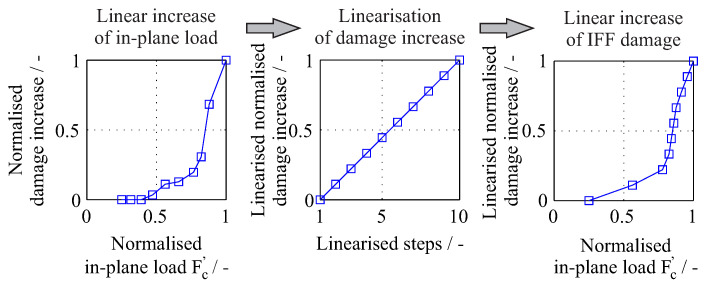
Illustration of the linear damage increase under in-plane loading.

Furthermore, the normalised in-plane load was used as a factor. However, in contrast to the experimental investigations [1], where a linear increase of the applied rotational velocity takes place, a linear increase of the resulting in-plane damage was simulated here. This was done through a linearisation of the damage increase under a non-linear increase of the applied in-plane load, as exemplarily shown in Figure 12. The selected factors for the out-of-plane as well as for the in-plane loading and their corresponding levels are shown in Table 1. The investigated damage sequences were generated based on a defined loading process. The initial damage was derived from previous experimental investigations [1] and was modelled as a distributed out-of-plane load at radius *r* and angle θ at the nodes included in the contact area between an impactor and the rotor [2]. The damage evolution was modelled as an in-plane load of rotational velocity steps of 1000 rpm in a range from 8000 rpm to 14,500 rpm.

For a representative investigation of the damage state and the dynamic behaviour of the composite rotors, in total 720 test cases were generated using a previously developed simulation tool [2]. For every calculated damage state, the failure modes for every layer at each element as well as the power spectral densities were calculated and extracted for the generation of the diagnostic database.

### 4.2. Labelling of Representative Damage Sequences

**Figure 13:**
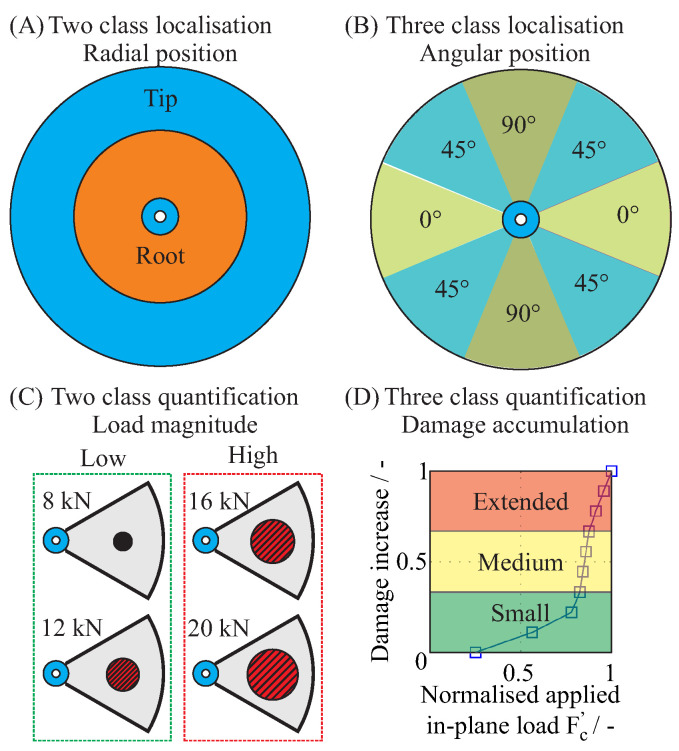
Four classifiers and their classification labels: for an out-of-plane load, a two class localisation (**A**), a three class localisation (**B**) and a two class load quantification (**C**); and, for an in-plane load, a three class damage accumulation (**D**).

The generated damage states were combined into one diagnostic database and the selected factors were assigned as labels for each damage state. Specifically, the position and the magnitude of the out-of-plane load as well as the in-plane load were assigned as labels, and were used for the classification of damage using different damage identification algorithms in Section 5 [15]. Using these labels, different independent data classes were formed, which contained similar structural conditions for different diagnostic levels. An overview of the different classes and their classification labels is shown in Figure 13.

The first classifier delivered the information about the radial location of the initial damage and was clustered in the two main distinguishable areas of the rotor, with a threshold radius of 150 mm. The classes were labelled “Rotor tip” and “Rotor root” to describe the location of the initial damage in the external area of the rotor or near the clamping area of the rotor, respectively. The second classifier distinguished between the angular position of the initial damage in the three typical directions for composites, and the classes were labelled as “$0^{\circ },45^{\circ },90^{\circ }$”. The third classifier distinguished between low and high magnitude out-of-plane loads, which were labelled “Low,High”, and the clustering was based on the experimental results [1]. Subsequently, for the inflicted damage from the in-plane load, a fourth class was defined based on the experimentally determined accumulated inter-fibre failure of the rotor and the classes were labelled as “Small, Medium, Extended”.

Each damage state in the diagnostic database then formed a tuple,

$$DD\left(i\right)=\left(S_{i},E_{i},\mathrm{Position},\mathrm{Angle},\mathrm{Load}\;\mathrm{magnitude},\mathrm{Damage}\;\mathrm{accumulation}\right),$$

where $S_{i}$ is the damage state, $E_{i}$ is the dynamic response, and “Position”, “Angle”, “Load magnitude”, and “Damage accumulation” are the corresponding labels.

## 5. Evaluation of Diagnostic Performance

For the performance estimation, different model validation techniques exist [79]. Here, the leave-one-out cross validation technique was selected, which is a model validation technique for assessing how the results of a statistical analysis can be generalised to an independent dataset, and how accurately a diagnostic model performs in practice. At each step of the leave-one-out cross validation, the dataset is partitioned in two subsets, the first subset containing classified sequences, and the second one containing an unclassified sequence, which is the test sequence. The procedure is repeated at every step using another sequence as the unclassified sequence, and the performance is calculated as the successful classifications over the total amount of steps. Compared to other techniques, e.g., bootstrapping and Monte Carlo cross validation, it reduces over-fitting and it guards against testing hypotheses that can be critical to the rotor, such as a “false positive”.

For the damage detection, i.e., Dagnostic Level 2, the performance evaluation was estimated using the experimental data, as they formed a well-defined feature space of the undamaged state. For the damage identification from in-plane and out-of-plane loading, i.e., Diagnostic Levels 2–4, the performance evaluation was estimated using the numerical data from the diagnostic database, as a detailed description of the expected damage states is required. Finally, for the damage identification and prediction, a performance comparison was conducted between conventional models, which were based on unsupervised learning techniques, and the developed sequence-based diagnostic model for an increasing amount of damage states.

### 5.1. Diagnostic Performance for Damage Detection, Level 1

The performance of the diagnostic model for damage detection is based, as described in Section 3.2, on the acquired experimental data for two statistic measures, the Kullback–Leibler divergence and the two-sample Kolmogorov–Smirnov test.

**Figure 14:**
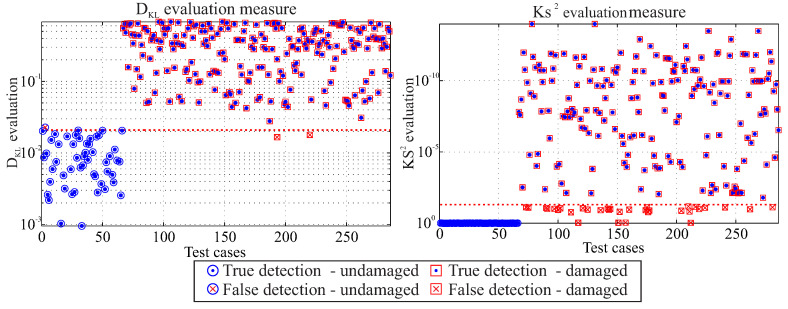
Diagnostic results under the null hypothesis for the investigated rotors for the Kullback–Leibler divergence $D_{KL}$ (**left**) and the two-sample Kolmogorov–Smirnov evaluation (**right**).

A total of 286 test cases were generated from the experimental data of the investigated rotors, where both the in-plane and the out-of-plane damage was considered [1]. The results from each test case, according to the statistical threshold, are presented in Figure 14, without any distinction between different damage types, as obviously only the existence or absence of damage is addressed in this level. Undamaged cases $E_{u}|S_{u}$ that are below the statistical threshold when compared to known undamaged cases $E_{0}|S_{0}$ are classified as true detections,

(38) $$\mathrm{True}\;\mathrm{detected}\;S_{0}={\left\{\begin{array}{ccc} 1 & \mathrm{if} & F_{Test}\left(E_{0},E_{u}\right)\leq l\\ 0 & \mathrm{else} \end{array}\right.}_{,}$$

whereas undamaged cases that are above the threshold as false detections. Similarly, damaged cases $E_{u}|S_{u}$ that are above the statistical threshold when compared to known damaged cases $E_{D}|S_{D}$ are classified as true detections,

(39) $$\mathrm{True}\;\mathrm{detected}\;S_{D}={\left\{\begin{array}{ccc} 1 & \mathrm{if} & F_{Test}\left(E_{D},E_{u}\right)>l\\ 0 & \mathrm{else} \end{array}\right.}_{,}$$

and damaged cases below the threshold as false ones. A mathematical expression is presented at Equation (40),

(40) $$\mathrm{Accuracy}\;\left(\%\right)=\frac{\mathrm{True}\;\mathrm{detected}\;S_{0}+\mathrm{True}\;\mathrm{detected}\;S_{D}}{\mathrm{Total}\;\mathrm{of}\;\mathrm{test}\;\mathrm{cases}}\cdot 100$$

A mean performance of 93.7% of all investigated cases was estimated for the damage detection from both statistical measures, as shown in Table 2.

A high amount of false detected cases “false negatives” is evident for the KS${}^{2}$ evaluation. This apparent decrease in the performance is due to the fact that the KS${}^{2}$ evaluation cannot clearly distinguish between undamaged cases and damaged cases from low magnitude in-plane loads, i.e., from 8000 rpm and 9000 rpm, given the whole investigated frequency range.

A careful selection of the investigated frequency range slightly changes the performance of the diagnostic level, making the tests more sensitive or more specific to damage detection. As the false prediction of damaged cases as undamaged ones is more critical than the undamaged cases detected as damaged ones, it seems that the $D_{KL}$ evaluation has an overall better accuracy based on the experimental data.

### 5.2. Sequence-Based Diagnostic Performance for Levels 2–4

A total of 72 different damage sequences each having 10 damage states were considered to estimate the performance of the developed sequence-based diagnostic model. For the identification of initial damage from out-of-plane load, a classification approach was selected to estimate the accuracy of the developed diagnostic model based on simulation data, as shown at Section 4.1. The similarity between each sequence and the total amount of known sequences was determined using the Viterbi algorithm.

**Figure 15:**
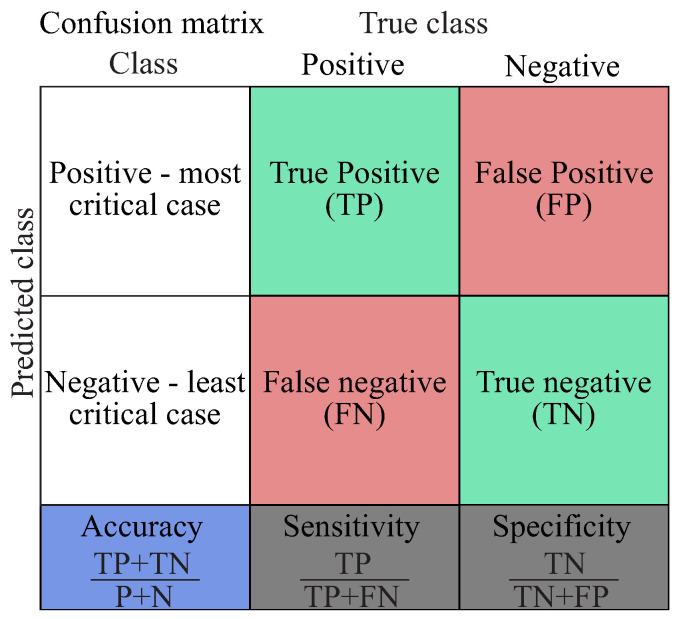
Illustration of a confusion matrix for the performance evaluation of a diagnostic model.

The diagnostic performance is presented as a confusion matrix in Figure 15. The numbers along the major diagonal (green) represent the correct predictions and the percentage of the correctly classified ones. The numbers of the off diagonal (red) represent the false predictions between the various classes [80]. The blue box on the bottom left of the matrix shows the overall accuracy of the classifier. The two grey boxes at the bottom of the matrix show the sensitivity and specificity, which indicate the percentage of positives and negatives, respectively, that are correctly predicted. As in many diagnostic cases, it is important to predict the most critical damage case and not to miss any, which means that with a high sensitivity of the diagnostic model, the probability of misclassifying the most critical damage case is reduced.

#### 5.2.1. Level 2: Identification of Initial Damage from Out-Of-Plane Load

**Figure 16:**
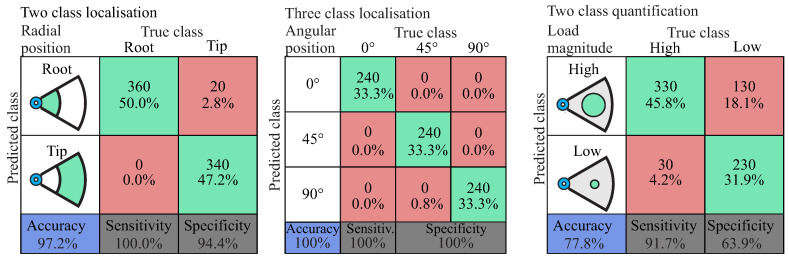
Diagnostic performance of classified damage cases regarding the radial and angular position as well as the magnitude of the out-of-plane load.

The performance estimation is shown in Figure 16 for three different classifiers regarding the radial and angular position as well as the magnitude of the out-of-plane load, with an estimated accuracy of 97.2%, 100% and 77.8%, respectively.

A perfect classification occurs for the angular position of the out-of-plane loading, which also indicates that the in-plane loading at different angles of a Cartesian orthotropic rotor has a distinct influence on the damage evolution sequence. In the two-class load magnitude quantification, a high number of false predictions is evident. This is due to the fact that the effect of initial damage from a high load in the tip of the rotor can have a similar effect as a low load in the root of the rotor, and thus misclassification can occur. However, a high sensitivity was estimated for all three classifiers, which for each classifier enables the correct classification of the most critical damage cases.

An accuracy of 77.8% was observed for the classifier of the load magnitude, which mainly suggests that the selection of the two classes “Low” and “High” load magnitude may not be optimal, and another class definition may be able to increase the diagnostic performance.

#### 5.2.2. Level 3: Identification of Propagating Damage from In-Plane Load

For the inflicted damage from the applied in-plane load, a three-class damage accumulation classifier was used. The first class, “small damage”, includes the first three damage states; the second class, “medium damage”, the States 4–6, and the third class; and “extensive damage”, the last four states of the investigated sequences.

**Figure 17:**
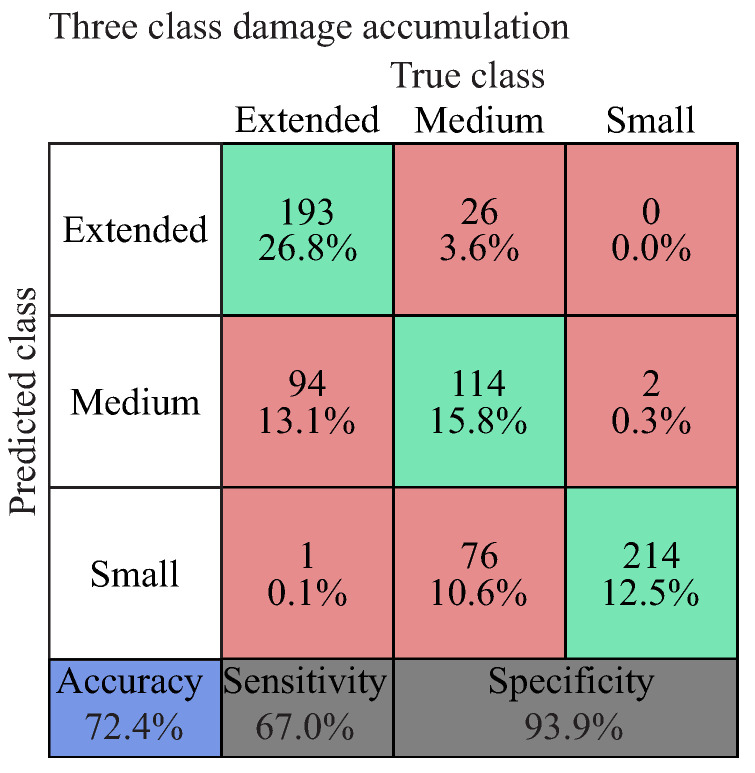
Diagnostic performance of the damage accumulation from an in-plane load.

The accuracy of the diagnostic model with regard to this classifier is shown in Figure 17. A high diagnostic accuracy was estimated of 72.4%. A higher sensitivity or higher specificity of each investigated class can be optimised by modifying the number of classes and the assigned damage states.

#### 5.2.3. Level 4: Prediction of Most Probable Damage Evolution

**Figure 18:**
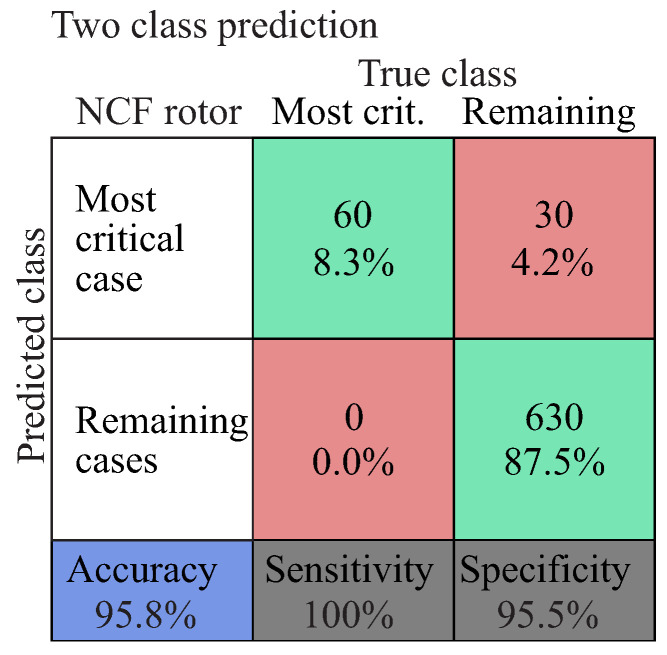
Diagnostic performance of the prediction of the most probable damage evolution.

To successfully predict the damage evolution, a precise identification of both the applied out-of-plane and in-plane load should be achieved. Therefore, a classifier with multiple classes was generated and, based on the previously identified classes for each rotor, a total of 36 classes were formed. The most critical case was then selected as the positive label in a two class problem, where its diagnostic performance is presented in Figure 18.

(41) $$\begin{array}{c} \mathrm{Most}\;\mathrm{critical}\;\mathrm{case}:\left\{\mathrm{Rotor}\;\mathrm{root},\;0{}^{\circ },\;\mathrm{High}\;\mathrm{load}\;\mathrm{magnitude},\;\mathrm{Extensive}\;\mathrm{damage}\;\mathrm{accumulation}\right\} \end{array}$$

Based on the two class classification of the prediction of the most critical damage case, a diagnostic accuracy of 95.8% was estimated. In the case where all 36 classes were considered for the damage prediction, a diagnostic accuracy of 76.4% was estimated. This performance decrease is due to the misclassification of neighbouring classes with similar damage states, which affects the diagnostic accuracy and the performance of the prediction classifier.

## 6. Discussion and Future Work

The contribution of the proposed damage identification method is the application and extension of sequence-based algorithms in the area of composite rotors, under consideration of their gradual damage behaviour and the corresponding structural dynamic response, in order to increase the structural performance of composite rotors. The diagnostic method provides a mean diagnostic performance above 70%, both for the damage detection from experimental data and for the damage identification and prediction of most probable damage evolution form simulation data. Two types of calculations were required for the proposed method. The first type of simulation was the Finite Element Analysis of the investigated composite rotors. The simulation was performed in a PC with an Intel(R) Core(TM) i7-2600 CPU at 3.8 GHz and 16 GB RAM. Each state of the rotor required approximately 6 min. The simulation time for the experiment presented in Section 4.1 and specifically for the total number of 720 test cases took approximately 74 h. For the evaluation of diagnostic performance presented in Section 5, the calculation time was approximately 80 min for both the damage detection and the damage identification, whereas 90% was for the testing and evaluating of the HMM.

Diagnostic models are generally a simplified representation of the reality, as they describe the relations between a small part of existing possible states and their outcomes. However, it is often impossible to even unequivocally establish these relations, due to a number of reasons. First, a well-defined relation is difficult to be conducted between damage increase and non-monotonic change of the dynamic behaviour. In addition to that, the analytical description of the physical processes are mostly inadequate, compelling an approximate solution using experimental data. Nevertheless, the experimental determination of a high number of states can be too complex, time consuming, cost intensive or even impossible. Especially for composite structures, the manufacturing-related features and the measurement errors add to the diagnostic uncertainty. In the case of an extension of the diagnostic database using data from simulation models, further uncertainties are included due to the inherent discrepancy between the simulation model and reality. All these considered, a more detailed performance estimation of the developed diagnostic models is required, in order to assess how these uncertainties influence the diagnostic output.

The results show that, to achieve a higher accuracy, the following measures should be undertaken. First, a higher number of experiments would result in a more profound statistical basis for the results. In the case of an extension of the diagnostic database using data from simulation models, a higher accordance between experimental and numerical results is very effective regarding the improvement of the simulation models and the use of advanced damage mechanics models. Additionally, the manufacturing-related uncertainties and deviations can be easily taken into account in the diagnostic method, if they are available.

## 7. Summary and Outlook

The present work provides a contribution to the area of vibration-based damage identification methods for composite rotors. By taking into consideration sequences of vibration responses, the proposed method can contribute to a vital improvement of safety and economic efficiency of composite rotors. This method should then be able to estimate the current structural condition as well as the most probable damage evolution scenario.

Based on the fundamental understanding of damage in composite rotors, a diagnostic system is then developed in which multiple sequences of the diagnostic features are analysed on four different diagnostic levels in order to increase the resolution of the damage identification. By using the validated model, input data are generated for the formation of a diagnostic database. The database is then applied for the development of the diagnostic models and for the evaluation of the diagnostic sensitivity and the overall performance. In general, the algorithms achieve a qualitative and quantitative separation of the investigated spectra, providing a high diagnostic performance.

A key benefit that further results from these investigations is the first steps towards a practical development approach for future high-performance rotors under special consideration of a realistic description of the damage-dependent structural dynamic behaviour. It is expected that the results will lead to a significantly improved understanding and evaluation of the damage-dependent dynamic behaviour of composite rotors under rotor-typical stress states and can subsequently be used in practice-oriented problems.

## Figures and Tables

**Table 1:** Selected factors for the out-of-plane loads, i.e., position and magnitude, and the rotational velocities.

Factor	Unit	Levels
out-of-plane load	kN	8, 12, 16, 20
radius, *r*	mm	75, 105, 135, 165, 195, 225
angle, θ	${}^{\circ }$	0, 45, 90
in-plane load	%	51, 53, 56, 58, 59, 60, 63, 69, 76, 100

**Table 2:** Performance estimation of the damage detection based on experimental data.

Method	Unit	Performance
KS${}^{2}$ evaluation accuracy	%	88.5
$D_{KL}$ evaluation accuracy	%	98.9
mean accuracy	%	93.7

## Abbreviations

The following abbreviations are used in this manuscript:

DES	damage evolution sequence
DRS	dynamic response sequence
PSD	power spectral density
RMS	root-mean-square
PDF	Probability density function
CDF	Cumulative distribution function
KS${}^{2}$-Test	two-sample Kolmogorov–Smirnov test
$D_{KL}$	Kullback–Leibler divergence
NCF	non-crimp fabric
HMM	hidden Markov model
